# Supplementary material for: A Likelihood Approach for Real-Time Calibration of Stochastic Compartmental Epidemic Models
Source: PLoS Comput Biol. 2017 Jan 17;13(1):e1005257. doi: 10.1371/journal.pcbi.1005257 (PMC5240920; doi:10.1371/journal.pcbi.1005257)
Supplement: S1 File — (TAR.GZ) [file pcbi.1005257.s014.tar.gz › HSPH_Online-SI-Revision/PF08/n100-extreme/n100-extreme_PF2_table4.pdf]

1	newly 3 weeks spec	2392.	3855.38	{3756., 3948.}	61.1781	0.207232	newly 3 weeks cumul	9902.	14 335.5	{14 213., 14 445.}	44.7742	0.160684	newly 5 weeks spec	1155.	2421.43	{2349., 2480.}	109.648	0.321384	newly 5 weeks cumul	12 790.	19 827.7	{19 674., 19 947.}	55.0253	0.190396	newly 50 weeks cumul	15 830.	27 945.3	{27 696., 28 229.}	76.5337	0.246814
2	newly 3 weeks spec	2435.	3359.75	{3263., 3442.}	37.9774	0.139729	newly 3 weeks cumul	9225.	12 221.5	{12 113., 12 334.}	32.4825	0.122145	newly 5 weeks spec	1337.	2248.93	{2176., 2296.}	68.2072	0.225755	newly 5 weeks cumul	12 421.	17 247.	{17 097., 17 379.}	38.8532	0.142549	newly 46 weeks cumul	16 331.	26 256.5	{26 082., 26 436.}	60.7772	0.206216
3	newly 3 weeks spec	1444.	3044.26	{2982., 3093.}	110.821	0.323863	newly 3 weeks cumul	6755.	12 083.8	{12 001., 12 160.}	78.8872	0.252572	newly 5 weeks spec	609.	1776.6	{1722., 1834.}	191.724	0.464827	newly 5 weeks cumul	8307.	16 198.3	{16 117., 16 282.}	94.9958	0.29002	newly 51 weeks cumul	9551.	21 643.7	{21 512., 21 802.}	126.612	0.355276
4	newly 3 weeks spec	766.	4225.73	{4130., 4321.}	451.662	0.741615	newly 3 weeks cumul	4576.	16 588.9	{16 463., 16 708.}	262.52	0.559324	newly 5 weeks spec	254.	2459.23	{2395., 2513.}	868.201	0.985891	newly 5 weeks cumul	5299.	22 279.5	{22 163., 22 393.}	320.446	0.623707	newly 55 weeks cumul	5634.	29 701.9	{29 587., 29 811.}	427.19	0.721965
5	newly 3 weeks spec	1978.	4017.49	{3942., 4092.}	103.109	0.307681	newly 3 weeks cumul	9313.	15 499.7	{15 402., 15 623.}	66.4306	0.221226	newly 5 weeks spec	894.	2418.63	{2366., 2482.}	170.54	0.432141	newly 5 weeks cumul	11 542.	21 053.8	{20 940., 21 197.}	82.4107	0.261046	newly 52 weeks cumul	13 327.	28 734.6	{28 600., 28 862.}	115.612	0.333669
6	newly 3 weeks spec	2370.	4260.54	{4180., 4336.}	79.7696	0.254669	newly 3 weeks cumul	9890.	15 593.8	{15 469., 15 712.}	57.6728	0.197749	newly 5 weeks spec	1132.	2711.12	{2620., 2794.}	139.498	0.379176	newly 5 weeks cumul	12 727.	21 714.3	{21 552., 21 878.}	70.6161	0.232014	newly 50 weeks cumul	15 600.	30 931.3	{30 725., 31 179.}	98.2772	0.297266
7	newly 3 weeks spec	1951.	4889.03	{4803., 4982.}	150.591	0.398917	newly 3 weeks cumul	8547.	16 530.9	{16 384., 16 650.}	93.4118	0.286475	newly 5 weeks spec	968.	3596.5	{3518., 3675.}	271.539	0.569935	newly 5 weeks cumul	10 776.	24 358.4	{24 211., 24 492.}	126.043	0.354186	newly 48 weeks cumul	12 929.	40 487.8	{40 175., 40 793.}	213.155	0.495752
8	newly 3 weeks spec	2287.	4178.14	{4106., 4249.}	82.6909	0.261677	newly 3 weeks cumul	9859.	15 273.1	{15 175., 15 366.}	54.9151	0.190088	newly 5 weeks spec	1178.	2697.06	{2620., 2768.}	128.952	0.359649	newly 5 weeks cumul	12 762.	21 336.	{21 191., 21 461.}	67.1837	0.223189	newly 50 weeks cumul	15 717.	30 664.5	{30 430., 30 913.}	95.1037	0.290258
9	newly 3 weeks spec	2292.	4801.24	{4725., 4900.}	109.478	0.321097	newly 3 weeks cumul	10 287.	16 954.6	{16 823., 17 053.}	64.8159	0.216993	newly 5 weeks spec	1160.	3230.58	{3151., 3309.}	178.498	0.444746	newly 5 weeks cumul	13 098.	24 165.1	{24 004., 24 311.}	84.4944	0.265978	newly 50 weeks cumul	15 554.	36 211.6	{35 956., 36 480.}	132.812	0.366999
10	newly 3 weeks spec	296.	3831.7	{3750., 3910.}	1194.49	1.11204	newly 3 weeks cumul	1966.	15 147.8	{15 042., 15 263.}	670.486	0.886758	newly 5 weeks spec	92.	2213.73	{2158., 2261.}	2306.23	1.38126	newly 5 weeks cumul	2237.	20 277.6	{20 164., 20 368.}	806.462	0.957346	newly 54 weeks cumul	2337.	26 947.1	{26 817., 27 047.}	1053.06	1.06185
11	newly 3 weeks spec	2110.	3207.12	{3123., 3281.}	51.9962	0.181766	newly 3 weeks cumul	9055.	12 072.8	{11 954., 12 195.}	33.3276	0.124908	newly 5 weeks spec	1137.	2002.35	{1942., 2055.}	76.1082	0.24566	newly 5 weeks cumul	11 827.	16 628.5	{16 473., 16 790.}	40.5976	0.147966	newly 48 weeks cumul	14 734.	23 501.3	{23 198., 23 861.}	59.5039	0.202743
12	newly 3 weeks spec	2018.	5189.17	{5107., 5270.}	157.144	0.410141	newly 3 weeks cumul	9115.	17 660.6	{17 517., 17 773.}	93.7527	0.287241	newly 5 weeks spec	959.	3728.1	{3643., 3816.}	288.749	0.589604	newly 5 weeks cumul	11 517.	25 824.2	{25 673., 25 982.}	124.227	0.350684	newly 49 weeks cumul	13 641.	41 629.1	{41 387., 41 872.}	205.176	0.484546
13	newly 3 weeks spec	3118.	4128.92	{4036., 4226.}	32.4221	0.121898	newly 3 weeks cumul	12 156.	14 108.2	{13 992., 14 213.}	16.0598	0.0646735	newly 5 weeks spec	2008.	3008.11	{2938., 3080.}	49.8063	0.175454	newly 5 weeks cumul	16 586.	20 652.3	{20 514., 20 774.}	24.5163	0.0952214	newly 47 weeks cumul	22 880.	34 217.2	{33 985., 34 436.}	49.5506	0.174783
14	newly 3 weeks spec	249.	6596.66	{6492., 6711.}	2549.26	1.42309	newly 3 weeks cumul	1934.	21 564.5	{21 399., 21 729.}	1015.02	1.04728	newly 5 weeks spec	37.	4852.18	{4746., 4962.}	13 014.	2.11768	newly 5 weeks cumul	2082.	32 153.2	{31 996., 32 310.}	1444.34	1.18874	newly 52 weeks cumul	2131.	51 460.6	{51 146., 51 754.}	2314.86	1.38289
15	newly 3 weeks spec	658.	3822.27	{3746., 3908.}	480.892	0.764041	newly 3 weeks cumul	3705.	14 837.8	{14 688., 15 011.}	300.479	0.602565	newly 5 weeks spec	197.	2275.15	{2211., 2337.}	1054.9	1.06245	newly 5 weeks cumul	4246.	20 066.8	{19 907., 20 248.}	372.605	0.67449	newly 52 weeks cumul	4525.	27 203.9	{27 038., 27 350.}	501.192	0.779008
16	newly 3 weeks spec	902.	4281.63	{4191., 4360.}	374.682	0.676354	newly 3 weeks cumul	5020.	16 585.5	{16 491., 16 676.}	230.388	0.51902	newly 5 weeks spec	342.	2549.67	{2487., 2608.}	645.518	0.872371	newly 5 weeks cumul	5877.	22 441.4	{22 332., 22 522.}	281.851	0.581891	newly 52 weeks cumul	6347.	30 335.3	{30 231., 30 452.}	377.946	0.679377
17	newly 3 weeks spec	2633.	3775.4	{3676., 3870.}	43.3878	0.156437	newly 3 weeks cumul	10 424.	13 605.6	{13 492., 13 716.}	30.5221	0.115674	newly 5 weeks spec	1461.	2549.	{2480., 2610.}	74.4695	0.241629	newly 5 weeks cumul	13 884.	19 274.5	{19 177., 19 374.}	38.8249	0.142463	newly 47 weeks cumul	18 108.	29 446.2	{29 206., 29 626.}	62.6144	0.211152
18	newly 3 weeks spec	1965.	4597.33	{4502., 4688.}	133.961	0.369096	newly 3 weeks cumul	8760.	14 824.6	{14 697., 14 939.}	69.2303	0.228469	newly 5 weeks spec	847.	3636.72	{3566., 3705.}	329.365	0.632775	newly 5 weeks cumul	10 903.	22 586.4	{22 432., 22 739.}	107.158	0.316294	newly 48 weeks cumul	12 657.	41 428.2	{41 180., 41 717.}	227.315	0.51496
19	newly 3 weeks spec	2260.	5264.05	{5165., 5347.}	132.923	0.367176	newly 3 weeks cumul	9867.	17 924.8	{17 784., 18 031.}	81.6641	0.259262	newly 5 weeks spec	1026.	3773.2	{3685., 3854.}	267.758	0.565499	newly 5 weeks cumul	12 362.	26 191.1	{26 026., 26 334.}	111.867	0.32606	newly 50 weeks cumul	14 680.	42 115.7	{41 912., 42 318.}	186.891	0.457714
20	newly 3 weeks spec	351.	5781.77	{5688., 5867.}	1547.23	1.21672	newly 3 weeks cumul	2657.	21 914.6	{21 812., 22 012.}	724.787	0.916338	newly 5 weeks spec	80.	3479.04	{3400., 3562.}	4248.8	1.6383	newly 5 weeks cumul	2903.	29 908.8	{29 792., 30 017.}	930.271	1.01295	newly 55 weeks cumul	3010.	40 712.6	{40 582., 40 829.}	1252.58	1.13116
21	newly 3 weeks spec	1266.	3777.43	{3686., 3847.}	198.375	0.474709	newly 3 weeks cumul	6452.	14 698.7	{14 585., 14 811.}	127.816	0.357578	newly 5 weeks spec	504.	2236.18	{2174., 2298.}	343.687	0.64699	newly 5 weeks cumul	7741.	19 840.8	{19 735., 19 937.}	156.307	0.408757	newly 52 weeks cumul	8578.	26 824.4	{26 705., 26 920.}	212.711	0.49514
22	newly 3 weeks spec	1097.	3559.79	{3480., 3635.}	224.502	0.511158	newly 3 weeks cumul	5662.	14 151.5	{14 036., 14 255.}	149.938	0.397824	newly 5 weeks spec	414.	2046.95	{1988., 2102.}	394.432	0.693989	newly 5 weeks cumul	6768.	18 908.1	{18 780., 19 023.}	179.375	0.446182	newly 53 weeks cumul	7400.	25 036.4	{24 860., 25 253.}	238.33	0.529333
23	newly 3 weeks spec	2274.	4201.72	{4121., 4288.}	84.7722	0.266582	newly 3 weeks cumul	9195.	13 849.3	{13 731., 13 969.}	50.6181	0.177869	newly 5 weeks spec	1192.	3257.98	{3185., 3329.}	173.32	0.436614	newly 5 weeks cumul	12 094.	20 837.5	{20 689., 20 980.}	72.296	0.236269	newly 46 weeks cumul	15 344.	37 697.8	{37 329., 37 988.}	145.685	0.390368
24	newly 3 weeks spec	2818.	4338.83	{4247., 4429.}	53.9684	0.18737	newly 3 weeks cumul	11 319.	14 154.4	{14 012., 14 287.}	25.0497	0.0970717	newly 5 weeks spec	1477.	3388.88	{3309., 3465.}	129.443	0.360601	newly 5 weeks cumul	14 831.	21 408.7	{21 266., 21 562.}	44.351	0.159414	newly 46 weeks cumul	19 249.	39 246.5	{38 921., 39 582.}	103.889	0.309384
25	newly 3 weeks spec	1110.	5464.62	{5379., 5537.}	392.308	0.692208	newly 3 weeks cumul	5663.	17 704.9	{17 559., 17 831.}	212.642	0.49504	newly 5 weeks spec	432.	4233.19	{4136., 4318.}	879.905	0.991126	newly 5 weeks cumul	6746.	26 813.8	{26 622., 26 981.}	297.476	0.599305	newly 49 weeks cumul	7332.	47 117.7	{46 830., 47 435.}	542.631	0.807957
26	newly 3 weeks spec	2919.	3580.81	{3488., 3660.}	22.6725	0.0886769	newly 3 weeks cumul	11 387.	13 076.4	{12 959., 13 197.}	14.8364	0.0600698	newly 5 weeks spec	1758.	2359.36	{2290., 2412.}	34.2071	0.127658	newly 5 weeks cumul	15 461.	18 351.6	{18 236., 18 472.}	18.6961	0.0744288	newly 47 weeks cumul	21 236.	27 347.1	{27 164., 27 552.}	28.7771	0.10983
27	newly 3 weeks spec	2411.	2971.23	{2881., 3066.}	23.2364	0.0905995	newly 3 weeks cumul	9503.	11 254.3	{11 122., 11 373.}	18.4288	0.073432	newly 5 weeks spec	1418.	1854.79	{1782., 1946.}	30.8032	0.116382	newly 5 weeks cumul	12 786.	15 470.4	{15 262., 15 662.}	20.9952	0.0827342	newly 48 weeks cumul	17 179.	21 754.2	{21 394., 22 167.}	26.6326	0.102486
28	newly 3 weeks spec	2066.	2894.55	{2829., 2964.}	40.1041	0.146384	newly 3 weeks cumul	9289.	11 581.4	{11 413., 11 753.}	24.6783	0.0957679	newly 5 weeks spec	1123.	1669.12	{1612., 1730.}	48.6305	0.171946	newly 5 weeks cumul	12 043.	15 457.4	{15 287., 15 665.}	28.3516	0.108382	newly 52 weeks cumul	14 773.	20 477.7	{20 246., 20 726.}	38.6154	0.141794
29	newly 3 weeks spec	2511.	4303.85	{4203., 4381.}	71.3998	0.233952	newly 3 weeks cumul	10 166.	14 236.2	{14 119., 14 340.}	40.0373	0.146236	newly 5 weeks spec	1397.	3304.06	{3217., 3377.}	136.511	0.373773	newly 5 weeks cumul	13 376.	21 338.	{21 176., 21 462.}	59.5241	0.202821	newly 46 weeks cumul	17 318.	38 099.4	{37 813., 38 400.}	119.999	0.342412
30	newly 3 weeks spec	1594.	4230.89	{4147., 4319.}	165.426	0.423892	newly 3 weeks cumul	7832.	16 560.4	{16 462., 16 655.}	111.446	0.325194	newly 5 weeks spec	651.	2469.25	{2406., 2531.}	279.301	0.578912	newly 5 weeks cumul	9505.	22 259.5	{22 173., 22 344.}	134.187	0.36956	newly 55 weeks cumul	10 576.	29 783.7	{29 657., 29 889.}	181.616	0.449656
31	newly 3 weeks spec	771.	4960.99	{4888., 5064.}	543.449	0.808477	newly 3 weeks cumul	4574.	19 233.2	{19 121., 19 354.}	320.489	0.623749	newly 5 weeks spec	189.	2925.8	{2855., 3000.}	1448.04	1.18971	newly 5 weeks cumul	5139.	25 984.7	{25 855., 26 105.}	405.637	0.703836	newly 56 weeks cumul	5392.	34 870.4	{34 734., 34 993.}	546.706	0.810705
32	newly 3 weeks spec	858.	3426.85	{3341., 3512.}	299.4	0.601336	newly 3 weeks cumul	4649.	13 603.7	{13 476., 13 714.}	192.615	0.466286	newly 5 weeks spec	288.	1976.43	{1907., 2042.}	586.26	0.836351	newly 5 weeks cumul	5438.	18 208.9	{18 111., 18 356.}	234.846	0.524839	newly 53 weeks cumul	5832.	24 207.9	{24 083., 24 403.}	315.088	0.618133
33	newly 3 weeks spec	3068.	3453.12	{3351., 3568.}	12.5528	0.0512265	newly 3 weeks cumul	12 287.	13 524.5	{13 262., 13 836.}	10.0719	0.0416147	newly 5 weeks spec	1670.	2036.65	{1942., 2117.}	21.9551	0.0859745	newly 5 weeks cumul	16 122.	18 231.7	{17 870., 18 665.}	13.0855	0.053343	newly 51 weeks cumul	20 713.	24 632.3	{24 077., 25 290.}	18.9217	0.0751786
34	newly 3 weeks spec	2614.	3618.2	{3545., 3687.}	38.4162	0.14113	newly 3 weeks cumul	10 179.	12 800.7	{12 668., 12 904.}	25.7556	0.0995131	newly 5 weeks spec	1528.	2508.86	{2439., 2575.}	64.1924	0.215247	newly 5 weeks cumul	13 712.	18 341.6	{18 183., 18 520.}	33.7628	0.126324	newly 46 weeks cumul	18 212.	28 919.	{28 545., 29 243.}	58.7906	0.200806
35	newly 3 weeks spec	2810.	4670.74	{4574., 4769.}	66.2185	0.220629	newly 3 weeks cumul	11 877.	17 011.6	{16 903., 17 116.}	43.2317	0.156034	newly 5 weeks spec	1428.	2971.07	{2906., 3037.}	108.058	0.31812	newly 5 weeks cumul	15 254.	23 741.3	{23 627., 23 877.}	55.6396	0.192116	newly 51 weeks cumul	18 586.	33 659.	{33 429., 33 854.}	81.0985	0.25791
36	newly 3 weeks spec	2377.	3005.19	{2911., 3092.}	26.4279	0.101739	newly 3 weeks cumul	9678.	11 573.2	{11 375., 11 748.}	19.5822	0.0776333	newly 5 weeks spec	1295.	1810.05	{1742., 1880.}	39.7722	0.145227	newly 5 weeks cumul	12 751.	15 728.1	{15 469., 15 972.}	23.348	0.0910949	newly 49 weeks cumul	16 300.	21 451.4	{21 021., 21 927.}	31.6038	0.119212
37	newly 3 weeks spec	918.	3879.93	{3806., 3956.}	322.65	0.625931	newly 3 weeks cumul	5030.	15 285.8	{15 180., 15 376.}	203.893	0.482715	newly 5 weeks spec	306.	2260.28	{2203., 2319.}	638.654	0.868335	newly 5 weeks cumul	5864.	20 505.1	{20 400., 20 593.}	249.678	0.543665	newly 54 weeks cumul	6382.	27 373.2	{27 270., 27 469.}	328.913	0.632367
38	newly 3 weeks spec	286.	5232.3	{5140., 5311.}	1729.48	1.26229	newly 3 weeks cumul	2079.	20 043.8	{19 921., 20 179.}	864.108	0.984121	newly 5 weeks spec	76.	3121.29	{3032., 3192.}	4006.96	1.61343	newly 5 weeks cumul	2297.	27 238.2	{27 105., 27 355.}	1085.82	1.07401	newly 54 weeks cumul	2347.	36 872.8	{36 754., 36 993.}	1471.06	1.19619
39	newly 3 weeks spec	1569.	4066.75	{3976., 4147.}	159.194	0.413567	newly 3 weeks cumul	6961.	14 825.9	{14 694., 14 938.}	112.985	0.32834	newly 5 weeks spec	719.	2671.52	{2598., 2748.}	271.561	0.569921	newly 5 weeks cumul	8738.	20 809.8	{20 675., 20 935.}	138.153	0.376851	newly 48 weeks cumul	10 021.	30 852.5	{30 620., 31 083.}	207.878	0.488371
40	newly 3 weeks spec	2069.	3025.82	{2926., 3121.}	46.2455	0.164934	newly 3 weeks cumul	8421.	11 869.	{11 646., 12 119.}	40.945	0.149002	newly 5 weeks spec	1045.	1782.37	{1714., 1869.}	70.5617	0.23161	newly 5 weeks cumul	10 908.	15 992.3	{15 627., 16 333.}	46.6107	0.166105	newly 49 weeks cumul	13 560.	21 482.	{20 941., 22 039.}	58.4218	0.199724
41	newly 3 weeks spec	1243.	2981.2	{2761., 3192.}	139.839	0.378866	newly 3 weeks cumul	5945.	11 741.6	{11 101., 12 438.}	97.5045	0.294903	newly 5 weeks spec	608.	1711.65	{1542., 1897.}	181.521	0.447772	newly 5 weeks cumul	7464.	15 723.	{14 822., 16 770.}	110.651	0.322748	newly 50 weeks cumul	8541.	20 687.3	{19 153., 22 411.}	142.212	0.382905
42	newly 3 weeks spec	457.	3855.55	{3776., 3931.}	743.665	0.926118	newly 3 weeks cumul	2671.	15 212.2	{15 100., 15 320.}	469.533	0.755512	newly 5 weeks spec	82.	2238.52	{2176., 2311.}	2629.9	1.43603	newly 5 weeks cumul	2987.	20 393.6	{20 278., 20 496.}	582.745	0.834255	newly 54 weeks cumul	3092.	27 157.2	{27 049., 27 273.}	778.306	0.943644
43	newly 3 weeks spec	2726.	4707.8	{4614., 4780.}	72.6999	0.237246	newly 3 weeks cumul	11 008.	14 633.6	{14 509., 14 755.}	32.936	0.123634	newly 5 weeks spec	1377.	3990.66	{3902., 4071.}	189.808	0.462047	newly 5 weeks cumul	14 359.	23 012.1	{22 836., 23 165.}	60.2626	0.204824	newly 47 weeks cumul	18 000.	46 702.3	{46 377., 47 022.}	159.457	0.41406
44	newly 3 weeks spec	1026.	3386.28	{3320., 3446.}	230.047	0.518523	newly 3 weeks cumul	5335.	13 433.4	{13 345., 13 526.}	151.797	0.401043	newly 5 weeks spec	393.	1953.75	{1889., 2008.}	397.137	0.696339	newly 5 weeks cumul	6350.	17 975.	{17 903., 18 055.}	183.071	0.451893	newly 53 weeks cumul	7041.	23 923.7	{23 812., 24 028.}	239.778	0.531192
45	newly 3 weeks spec	374.	4232.05	{4152., 4304.}	1031.56	1.05364	newly 3 weeks cumul	2546.	16 415.3	{16 301., 16 532.}	544.747	0.809383	newly 5 weeks spec	135.	2514.25	{2453., 2584.}	1762.41	1.26999	newly 5 weeks cumul	2913.	22 208.6	{22 103., 22 291.}	662.396	0.882178	newly 52 weeks cumul	3058.	30 006.7	{29 884., 30 133.}	881.253	0.991779
46	newly 3 weeks spec	1322.	4503.64	{4427., 4588.}	240.669	0.532296	newly 3 weeks cumul	7028.	17 203.8	{17 080., 17 312.}	144.79	0.388788	newly 5 weeks spec	520.	2713.34	{2647., 2766.}	421.796	0.717418	newly 5 weeks cumul	8416.	23 432.1	{23 296., 23 541.}	178.423	0.444702	newly 52 weeks cumul	9218.	32 117.8	{31 988., 32 256.}	248.425	0.542107
47	newly 3 weeks spec	877.	3819.98	{3732., 3907.}	335.574	0.638998	newly 3 weeks cumul	5116.	15 087.6	{14 973., 15 204.}	194.91	0.469682	newly 5 weeks spec	280.	2219.05	{2164., 2279.}	692.518	0.89892	newly 5 weeks cumul	5861.	20 221.	{20 102., 20 334.}	245.009	0.537824	newly 54 weeks cumul	6385.	26 917.9	{26 757., 27 057.}	321.58	0.624876
48	newly 3 weeks spec	1336.	4610.97	{4527., 4697.}	245.132	0.537942	newly 3 weeks cumul	7014.	18 020.	{17 897., 18 127.}	156.915	0.409783	newly 5 weeks spec	498.	2701.71	{2634., 2757.}	442.512	0.734345	newly 5 weeks cumul	8264.	24 277.1	{24 156., 24 395.}	193.769	0.468003	newly 54 weeks cumul	8948.	32 424.7	{32 320., 32 539.}	262.368	0.559148
49	newly 3 weeks spec	215.	5058.64	{4978., 5146.}	2252.86	1.37156	newly 3 weeks cumul	1583.	19 592.2	{19 456., 19 730.}	1137.66	1.0926	newly 5 weeks spec	43.	2993.8	{2918., 3061.}	6862.33	1.84269	newly 5 weeks cumul	1735.	26 493.3	{26 345., 26 610.}	1426.99	1.18383	newly 56 weeks cumul	1768.	35 619.6	{35 522., 35 733.}	1914.68	1.3042
50	newly 3 weeks spec	2185.	3940.42	{3862., 4034.}	80.3396	0.256036	newly 3 weeks cumul	9176.	13 926.1	{13 807., 14 037.}	51.7668	0.181169	newly 5 weeks spec	1236.	2722.9	{2649., 2792.}	120.299	0.342929	newly 5 weeks cumul	12 158.	19 941.7	{19 810., 20 059.}	64.0212	0.214895	newly 47 weeks cumul	15 907.	31 350.8	{31 158., 31 562.}	97.0882	0.294655
